# Integrated RNA-seq and small RNA-seq profiling reveals tissue-associated transcriptional programs and candidate miRNA–mRNA relationships across tea floral tissues

**DOI:** 10.3389/fpls.2026.1941110

**Published:** 2026-09-14

**Authors:** Feng Xing, Feipeng Sun, Qingying Xie, Tianyu Li, Jinyan Yang, Shuangping Heng, Ling Xu, Wei Zhang, Guangzhi Mao

**Affiliations:** 1 College of Life Sciences, Xinyang Normal University, Xinyang, Henan, China; 2 Chinese Academy of Medical Sciences and Peking Union Medical College, Beijing, China

**Keywords:** Camellia sinensis, floral tissue differentiation, miRNA–mRNA relationships, RNA-Seq, small RNA-seq, tea flower, WGCNA

## Abstract

**Introduction:**

Tea flowers comprise anatomically and functionally specialized tissues, yet the transcriptional and post-transcriptional programs that distinguish these tissues remain poorly characterized.

**Methods:**

In this study, flower buds of *Camellia sinensis* cv. ‘Fudingdabaicha’ at the bud-white stage were dissected into seven tissues: ovary, outer green petal, inner white petal, filament, sepal, anther, and style. RNA-seq and small RNA-seq libraries were prepared from the same 21 biological samples, enabling direct comparison and integration of mRNA and miRNA expression profiles.

**Results:**

Across the 21 pairwise tissue comparisons, 33,333 nonredundant differentially expressed genes were identified, revealing substantial variation in transcriptomic divergence among tissues. The outer green and inner white petals had the most similar transcriptomic profiles, and the genes that distinguished them were enriched mainly in plastid-associated and photosynthetic processes. By contrast, the anther and style showed the greatest transcriptomic divergence, involving metabolic, structural, and signaling functions. Weighted gene co-expression network analysis identified 24 modules, including three representative modules whose eigengenes showed positive correlations with the anther, sepal, and style traits. These modules were enriched in processes related to pollen germination and polarized growth, cell wall remodeling and organ growth, and RNA metabolism and chromatin-associated developmental regulation, respectively. RT-qPCR analysis of six representative genes showed tissue expression patterns broadly concordant with the RNA-seq data. Small RNA profiling identified 215 miRNAs, including 54 homologs of known plant miRNA families and 161 putative novel miRNAs; 175 miRNAs were differentially expressed among tissues. Intersecting psRNATarget predictions with candidate hub genes from the three representative modules yielded 45 candidate miRNA–mRNA relationships involving 39 miRNAs and 28 candidate hub genes. Six representative predicted pairs showed significant negative correlations across the matched samples.

**Discussion:**

These findings characterize tissue-dependent mRNA and miRNA expression programs in tea flowers and prioritize candidate genes and miRNA–mRNA pairs for functional studies of floral tissue differentiation and reproductive development.

## 1 Introduction

The tea plant (*Camellia sinensis*) is an important perennial evergreen woody crop whose young shoots and leaves are harvested for tea production and contain metabolites that determine tea flavor and quality (Chen et al., 2023). In addition to vegetative growth, tea plants undergo a prolonged reproductive cycle. Floral bud differentiation and floral organ development extend over several months, and vegetative buds and flower buds at different developmental stages may coexist on the same plant, resulting in complex coordination between vegetative and reproductive growth (Tan et al., 2013; Chen et al., 2018). Although commercial tea production depends primarily on sustained vegetative shoot growth, flowering and fruiting are essential for sexual reproduction, maintenance of genetic diversity, and hybrid breeding. Developing flowers and fruits also act as reproductive sinks and alter the allocation of carbon, nitrogen, and mineral nutrients between reproductive and vegetative organs (Tan et al., 2024). Tea flowers contain flavonoids, polyphenols, tea saponins, polysaccharides, aroma-related compounds, and other bioactive constituents, making them a potentially valuable secondary resource (Chen et al., 2018; Tang et al., 2022; Gao et al., 2023). The abundance of taste- and aroma-related metabolites also varies across reproductive stages of tea flowers (Shi et al., 2025). Characterizing the molecular programs underlying floral tissue differentiation is therefore relevant to both tea reproductive biology and the potential utilization of tea flowers.

A flower comprises organs and tissues that differ in morphology, cellular composition, and physiological function. Sepals form the outermost floral whorl and protect the developing inner organs, whereas petals contribute to corolla formation and pollinator attraction. The identities of the major floral organs are established through conserved developmental regulatory programs (Coen and Meyerowitz, 1991). Filaments support the anthers and facilitate nutrient transport, and anthers are responsible for pollen formation, maturation, and storage. The style connects the stigma to the ovary and contains the transmitting tract through which pollen tubes grow toward the ovules, whereas the ovary encloses the ovules and subsequently supports seed and fruit development after fertilization (Broz and Bedinger, 2021; Pereira et al., 2021). Although these tissues arise within the same flower, each must establish distinct programs of cell proliferation, transport, signaling, cytoskeletal organization, cell wall modification, and metabolism to perform its specialized functions. Transcriptomic analyses of floral organs have revealed pronounced tissue-dependent expression patterns, including organ-preferentially expressed genes and miRNA–target regulatory modules (Yang et al., 2021). Sequencing whole flowers or mixtures of floral organs can therefore mask expression differences among reproductive tissues such as anthers, styles, and ovaries. Dissection and independent profiling of tissues collected at the same developmental stage provide a more direct means of resolving floral tissue specialization and reproductive expression programs.

Previous molecular studies of tea flowers have mainly examined mixed floral organs, developmental time courses, or abnormal reproductive phenotypes. Transcriptome sequencing of mixed petals, stamens, and pistils generated floral sequence resources and supported molecular marker development in tea plant (Tan et al., 2013). Subsequent studies identified genes and transcription factors associated with floral induction, organ differentiation, and developmental progression (Liu et al., 2017, 2020a), and revealed changes in auxin, gibberellin, abscisic acid, and ethylene signaling during tea flower development (Xu et al., 2022). Comparisons of pistil-deficient and male-sterile flower buds further identified candidate genes and miRNAs associated with reproductive organ formation and floral sterility (Liu et al., 2020b; Qu et al., 2021). More recently, functional characterization of *CsSTK* and its upstream regulator *CsBPC5* provided direct evidence for their roles in tea floral organ and pistil development (Pang et al., 2025). Despite these advances, matched mRNA and small RNA profiles from individually dissected tissues of normal tea flower buds have not been systematically integrated.

Differential expression analysis identifies genes whose expression varies among tissues, whereas co-expression analysis groups genes with coordinated expression patterns. Weighted gene co-expression network analysis (WGCNA) provides a framework for defining such gene modules (Langfelder and Horvath, 2008) and has been used to identify modules associated with catechin, theanine, and caffeine biosynthesis in tea plant (Tai et al., 2018). Plant miRNAs regulate floral development and reproduction through target mRNA cleavage, translational repression, and interactions with developmental gene regulatory networks (Arnaud and Laufs, 2023). Well-characterized examples include the miR156–*SPL*, miR172–*AP2*, miR319–*TCP*, and miR396–*GRF* modules, which regulate developmental phase transitions, floral organ identity, petal and stamen growth, and cell proliferation, respectively (Chen, 2004; Jones-Rhoades et al., 2006; Nag et al., 2009; Wu et al., 2009; Yang et al., 2021). Because sequence-based target prediction commonly produces large candidate sets, integration with matched mRNA expression data and tissue-associated co-expression modules can help prioritize biologically relevant miRNA–mRNA relationships (Axtell and Meyers, 2018; Dai et al., 2018; Zhang et al., 2020; Zheng et al., 2024).

In this study, flower buds of *Camellia sinensis* cv. ‘Fudingdabaicha’ at the bud-white stage were dissected into seven anatomically distinct tissues: ovary, outer green petal, inner white petal, filament, sepal, anther, and style. Matched RNA-seq and small RNA-seq profiles were generated from the same 21 tissue samples. The study had three aims. First, we characterized tissue-associated mRNA and miRNA expression profiles. Second, we identified co-expression modules and candidate hub genes associated with anther, sepal, and style functions. Third, we prioritized predicted miRNA–mRNA relationships for further study. These analyses provide a tissue-resolved view of mRNA and miRNA expression patterns across tea floral tissues.

## 2 Materials and methods

### 2.1 Plant materials and sample collection

*Camellia sinensis* cv. ‘Fudingdabaicha’, a nationally registered tea cultivar in China (GS13001-1985), was used in this study. The experimental site was located at the Xiangyun Tea Base in Xinyang, Henan Province, China (114.09°E, 32.15°N). The site has a mean annual rainfall of 1,100 mm, a mean annual temperature of 15.3 °C, a mean annual relative humidity of 77%, a frost-free period of 225 days, and an average annual sunshine duration of 2,000 h. Flower buds at the bud-white stage were collected between 09:00 and 11:00 on 15 November 2021. At this stage, the buds had a milky-white, closed corolla; the anthers had not dehisced, and pollen grains remained within the anther locules. Healthy and undamaged flower buds were collected from 12 tea plants of similar age grown under uniform field management conditions. On each plant, three to four flower buds were sampled from each of the upper, middle, and lower canopy layers. Different canopy orientations were represented to reduce positional effects. A total of 120 flower buds collected from the 12 tea plants were transported to the laboratory in a chilled container. All buds were combined and randomly allocated to three non-overlapping groups before dissection. After allocation, the three groups were dissected and processed separately. Within each group, flower buds were dissected on ice using sterile forceps and scissors into seven tissues: ovary (OV), outer green petal (OG), inner white petal (IW), filament (FI), sepal (SE), anther (AN), and style (ST) (**Figure 1**). The outer green petals and inner white petals represented two anatomically distinct petal layers within the closed corolla. Within each group, tissues of the same type were pooled across all dissected flower buds to form one pooled biological replicate. Within each group, each tissue pool underwent separate RNA extraction, library construction, and sequencing. The experimental unit was one non-overlapping pool of flower buds. The three replicate pools were derived from the same set of 12 plants, resulting in three pooled biological replicates per tissue and 21 samples in total. All tissues were dissected within 2 h and immediately frozen at −80 °C. The frozen samples were shipped on dry ice from Xinyang to the sequencing service provider in Wuhan on the same day. They arrived on the following day, and total RNA extraction was initiated upon arrival.

**Figure 1:**
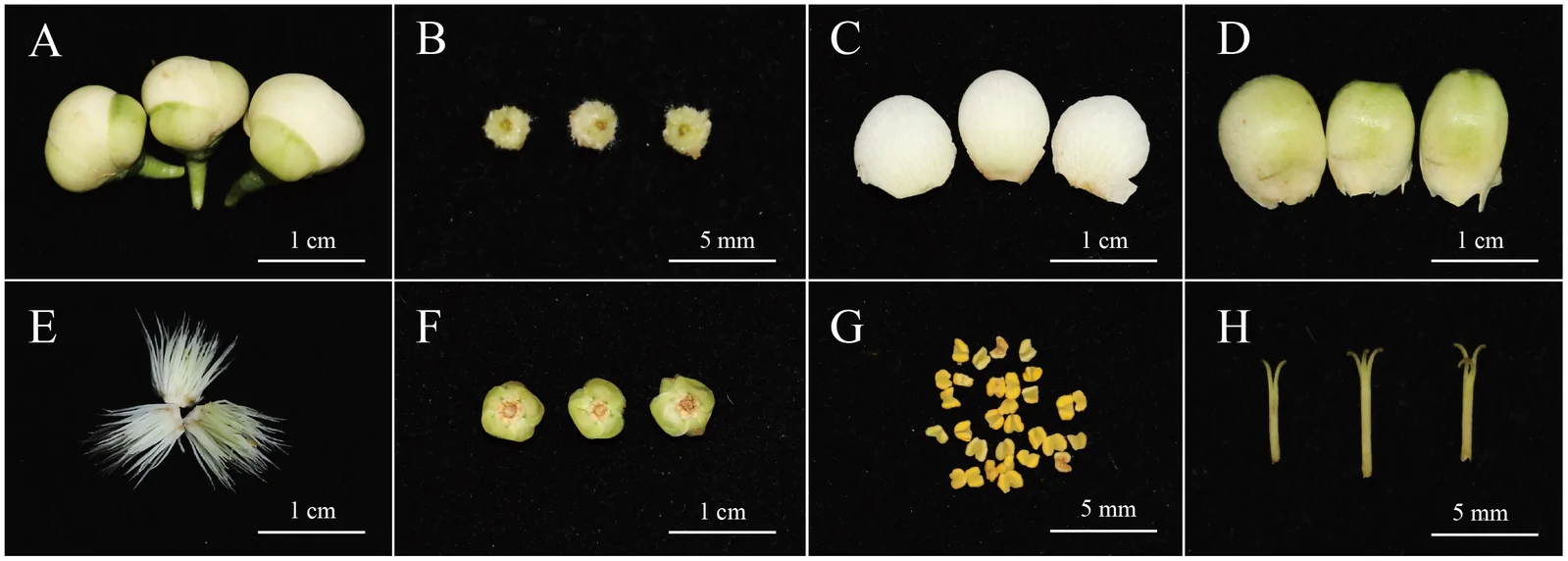
Representative images of tea flower buds and dissected floral tissues at the bud-white stage. **(A)** Flower buds at the bud-white stage. **(B)** Ovary (OV). **(C)** Inner white petal (IW). **(D)** Outer green petal (OG). **(E)** Filament (FI). **(F)** Sepal (SE). **(G)** Anther (AN). **(H)** Style (ST). Scale bars: 1 cm in **(A)** and **(C–F)**; 5 mm in **(B, G, H)**.

### 2.2 Total RNA extraction, library preparation, and sequencing

Total RNA was extracted from each floral tissue sample using RNA isolater Total RNA Extraction Reagent (Vazyme, Nanjing, China; R401-01) according to the manufacturer’s instructions. RNA integrity was assessed using an Agilent 2100 Bioanalyzer with the RNA 6000 Pico Kit (Agilent Technologies, 5067-1513). Only RNA samples with an RNA integrity number (RIN) ≥ 7.0 and no evidence of degradation or contamination were used for library construction. Aliquots of the same total RNA preparation from each biological sample were used for small RNA sequencing and RNA sequencing, ensuring one-to-one correspondence between the small RNA-seq and RNA-seq libraries.

#### 2.2.1 Small RNA library construction and sequencing

Small RNA libraries were constructed from 0.1–1 μg of total RNA using the VAHTS™ Small RNA Library Prep Kit for Illumina^®^ (Vazyme, Nanjing, China; NR801) according to the manufacturer’s instructions. Adapter ligation, reverse transcription, PCR amplification, product purification, and size selection were performed using the supplied reagents. Library quality and fragment-size distribution were assessed using an Agilent 2100 Bioanalyzer. Qualified small RNA libraries were sequenced on the Illumina NovaSeq 6000 platform using a single-end 150-bp sequencing strategy.

#### 2.2.2 RNA-seq library construction and sequencing

For RNA-seq library construction, 1–5 μg of total RNA from each sample was subjected to ribosomal RNA depletion using the Ribo-off^®^ rRNA Depletion Kit (Plant) (Vazyme, Nanjing, China; N409). Libraries were constructed from the rRNA-depleted RNA using the VAHTS Universal V8 RNA-seq Library Prep Kit for Illumina^®^ (Vazyme, Nanjing, China; NR605) according to the manufacturer’s instructions. Following RNA fragmentation and standard cDNA library preparation, library quality and fragment-size distribution were assessed using an Agilent 2100 Bioanalyzer. Qualified RNA-seq libraries were sequenced on the Illumina NovaSeq 6000 platform using a paired-end 150-bp sequencing strategy.

### 2.3 RNA-seq data processing and gene expression quantification

Raw paired-end RNA-seq reads were assessed using FastQC v0.12.1 and processed using fastp v0.21.0 to remove adapter contamination, low-quality reads, and reads containing ambiguous bases. Clean reads were aligned to the *Camellia sinensis* cv. ‘Shuchazao’ v2 reference genome (Xia et al., 2020) using HISAT2 v2.1.0 (Kim et al., 2019). The reference genome file shuchazao_V2.genome.fas.gz and the corresponding gene annotation file shuchazao_V2.gff3.gz were downloaded from the Tea Plant Information Archive in March 2023 (TPIA; Gao et al., 2024; https://tpia.teaplants.cn/download.html). At the time of reference selection in March 2023, a cultivar-specific high-quality genome assembly with matched gene annotation was not available for ‘Fudingdabaicha’. The ‘Shuchazao’ v2 assembly was selected because it provides a high-quality chromosome-level reference and a matched annotation system for *Camellia sinensis* var. *sinensis* (Xia et al., 2020). ‘Fudingdabaicha’ and ‘Shuchazao’ both belong to *C. sinensis* var. *sinensis*. The same reference coordinate system and gene identifiers were used throughout RNA-seq and small RNA-seq alignment, expression quantification, miRNA identification, target prediction, WGCNA, and integrated analysis. Alignment files were converted to BAM format and sorted using SAMtools v1.21. Transcript abundance was quantified using StringTie v2.1.2 in reference annotation-only mode, without novel transcript assembly (Pertea et al., 2015). Gene- and transcript-level count matrices were generated from the StringTie outputs using prepDE.py with an average read length of 150 bp, and gene expression levels were expressed as fragments per kilobase of transcript per million mapped fragments (FPKM). Principal component analysis and Pearson correlation analysis were performed using log_2_(FPKM + 1)-transformed expression values of all quantified genes. For Euclidean distance analysis, raw counts were filtered using filterByExpr in edgeR v4.8.1, normalized using the trimmed mean of M values (TMM) method (Robinson et al., 2010; Robinson and Oshlack, 2010), and converted to counts per million (CPM). CPM values were averaged across the three biological replicates within each tissue and then standardized across the seven tissues for each gene before calculating pairwise Euclidean distances.

### 2.4 Differential gene expression and gene ontology enrichment analyses

Differential gene expression analysis was performed in R v4.3.1 using DESeq2 (Love et al., 2014). Genes with a mean raw count of 1 or lower were excluded. The DESeq2 design formula included tissue as the explanatory factor, and all 21 pairwise comparisons among the seven tissues were performed. Genes with |log_2_ fold change| ≥ 1 and a Benjamini–Hochberg-adjusted *P* value < 0.05 were considered differentially expressed. DEGs identified in the 21 comparisons were combined and deduplicated to generate the nonredundant DEG set used for WGCNA and downstream enrichment analyses.

Gene Ontology enrichment analysis was performed using the enricher function in clusterProfiler v4.14.4 (Wu et al., 2021). GO annotations for the ‘Shuchazao’ v2 gene models were obtained from the shuchazao2_annotation.zip package. The package was downloaded from TPIA in March 2023 (https://tpia.teaplants.cn/download.html). All annotated tea plant genes were used as the background set. Biological Process (BP), Cellular Component (CC), and Molecular Function (MF) terms with a Benjamini–Hochberg-adjusted *P* value < 0.05 were considered significantly enriched. The same background set was used for the enrichment analyses of pairwise DEGs, WGCNA modules, and candidate hub gene sets.

### 2.5 Weighted gene co-expression network analysis and candidate hub gene identification

Weighted gene co-expression network analysis was performed using WGCNA v1.73 (Langfelder and Horvath, 2008) and the untransformed FPKM expression matrix of the nonredundant DEGs. All 21 libraries were generated and processed using the same workflow. WGCNA evaluates gene-wise expression correlations across samples. For a given gene, the transcript length factor is constant across the 21 samples and does not alter its Pearson correlations with other genes. The consistently processed FPKM matrix was therefore used directly for network construction. Genes and samples were checked using goodSamplesGenes and hierarchical sample clustering, and all 21 samples were retained. An unsigned Pearson correlation network was constructed using a soft-thresholding power of 7. The adjacency matrix was converted into a topological overlap matrix (TOM), and genes were clustered using TOM-based dissimilarity. Modules were identified using dynamic tree cutting with a minimum module size of 30, deepSplit = 2, and pamRespectsDendro = FALSE. Modules with eigengene dissimilarity below 0.25 were merged. Module eigengenes were correlated with binary tissue traits using Pearson correlation analysis.

Module membership (MM) was calculated as the correlation between each gene and its module eigengene, whereas gene significance (GS) was calculated as the correlation between each gene and the corresponding tissue trait (Langfelder and Horvath, 2008). Candidate hub gene identification, GO enrichment analysis, and network visualization were subsequently performed for the turquoise, red, and yellow modules. Candidate hub genes were required to meet four criteria: MM ≥ 0.80, GS ≥ 0.30, the highest mean expression in the corresponding tissue among the seven tissues, and intramodular connectivity within the top 10% of the respective module. These four criteria were used as a study-specific prioritization strategy. MM ≥ 0.80 retained genes with strong module membership, and GS ≥ 0.30 retained genes with a positive association with the corresponding tissue trait. The tissue-maximum expression criterion added tissue preference. The top 10% connectivity criterion prioritized relatively central genes within each module and provided a consistent percentile-based filter across modules of different sizes. Genes meeting all four criteria were described as candidate hub genes. Intramodular connectivity was calculated using all genes and edges in each module before applying the TOM thresholds used for network visualization. GO enrichment analysis was further performed on the identified candidate hub gene sets. For network visualization, mRNA–mRNA edges with TOM weights greater than 0.70, 0.47, and 0.50 were retained for the turquoise, red, and yellow modules, respectively. The resulting networks were visualized using Cytoscape v3.10.1.

### 2.6 Reverse-transcription quantitative PCR validation

Six representative genes were selected for RT-qPCR validation. Gene-specific primers were designed using Primer Premier v5.0, and their sequences are listed in **Supplementary Table 3**. First-strand cDNA was synthesized from 1 μg of total RNA using the PrimeScript RT Reagent Kit with gDNA Eraser. Genomic DNA was removed at 42 °C for 2 min, followed by reverse transcription at 37 °C for 15 min and enzyme inactivation at 85 °C for 5 s. The resulting cDNA was diluted fivefold before quantitative PCR.

Quantitative PCR was performed using a SYBR Green-based qPCR master mix (TIANGEN, Beijing, China) on a CFX96 Real-Time PCR Detection System (Bio-Rad, Hercules, CA, United States) in a total reaction volume of 10 μL. The amplification program consisted of 95 °C for 4 min, followed by 40 cycles of 94 °C for 15 s and 60 °C for 15 s. Melting-curve analysis was conducted to verify amplification specificity. The *β-actin* gene was used as the internal reference, and relative expression was calculated using the 2^−ΔΔCt^ method, with OV used as the calibrator. Three biological replicates were analyzed for each tissue, and each biological replicate was measured in three technical replicates. The three technical measurements were averaged for each biological replicate, and the mean and SD were calculated from the three biological replicates.

### 2.7 Small RNA data processing, miRNA identification, and expression analysis

Raw single-end 150-bp small RNA reads were processed using fastp v0.21.0 to remove adapter sequences, low-quality reads, and reads containing ambiguous bases. The quality of the processed reads was assessed using FastQC v0.12.1. Clean reads were aligned to the *Camellia sinensis* cv. ‘Shuchazao’ v2 reference genome using Bowtie2 v2.4.2 (Langmead and Salzberg, 2012). Candidate miRNAs were identified independently in each library using miRDeep-P2 (miRDP2) v1.1.4 (Kuang et al., 2019). Sequences derived from other classes of noncoding RNA were filtered using Rfam release 14.8 (Kalvari et al., 2021). The Rfam data were downloaded in March 2023 from https://ftp.ebi.ac.uk/pub/databases/Rfam/14.8/. Precursor secondary structures were evaluated using ViennaRNA v2.5.1. Predictions located on the same chromosome and sharing the same mature miRNA start coordinate were merged into a single nonredundant miRNA locus.

Representative mature miRNA sequences were searched against mature plant miRNAs in miRBase release 22 using BLASTN v2.16.0 with the blastn-short task (Kozomara et al., 2019). The mature.fa file was downloaded from the miRBase release 22 archive in March 2023 (https://www.mirbase.org/download/PREVIOUS_RELEASES/22). Alignments with no more than one nucleotide mismatch and an E-value < 0.05 were retained. Candidates with qualifying matches were classified as homologs of known plant miRNA families, whereas those without qualifying matches were classified as putative novel miRNAs.

Reads mapping to the 215 nonredundant mature miRNA loci were quantified using featureCounts v2.0.6 in single-end mode at the meta-feature level, with a minimum overlap of 1 bp (Liao et al., 2014). Only primary alignments were considered; multi-mapping reads were included, whereas reads assigned to multiple overlapping features were excluded. Transcripts per million (TPM) values were used for expression visualization, and variance-stabilized count data were used for principal component analysis and Pearson correlation analysis. Mature miRNA length and 5′ terminal nucleotide distributions were summarized from the representative sequences. To summarize the tissue-level recovery of the identified miRNA loci, a miRNA was considered recovered in a tissue when it was predicted by miRDP2 in at least one of the three libraries from that tissue. A binary presence–absence matrix was generated from the source-library information, and an UpSet plot was used to visualize miRNA loci recovered from individual tissues or shared among multiple tissues.

Differential miRNA expression analysis was performed using the raw integer count matrix in DESeq2, with tissue as the explanatory factor and the poscounts method used for size-factor estimation. All 21 pairwise comparisons among the seven tissues were performed. MiRNAs with |log_2_ fold change| ≥ 1 and a Benjamini–Hochberg-adjusted *P* value < 0.05 were considered differentially expressed.

### 2.8 miRNA target prediction and integrated network analysis

Potential targets of the identified miRNAs were predicted using psRNATarget (Dai et al., 2018; https://www.zhaolab.org/psRNATarget/), accessed on 22 January 2025. Mature miRNA sequences were used as queries. Coding sequences from the shuchazao_V2_CDS.fas.gz file were used as the target dataset. The CDS file was downloaded from TPIA in March 2023 (https://tpia.teaplants.cn/download.html). Schema V2 was applied with a maximum expectation value of 5. All remaining parameters were retained at their default settings. Duplicate predictions for the same miRNA–mRNA pair were removed, and the record with the lowest expectation value was retained.

Predicted target genes were intersected with candidate hub genes from the turquoise, red, and yellow modules. For each module, the TOM-derived mRNA co-expression network described in Section 2.5 was retained, and psRNATarget-predicted miRNA–mRNA edges were added to construct the integrated network. The resulting networks were visualized using Cytoscape v3.10.1. Spearman’s rank correlation coefficients were calculated between miRNA TPM values and mRNA FPKM values across the 21 matched samples, and *P* values were adjusted using the Benjamini–Hochberg method. Six representative predicted pairs with *ρ* ≤ −0.60 and an adjusted *P* value < 0.05 were selected, with no miRNA or mRNA repeated among the six pairs.

## 3 Results

### 3.1 RNA-seq data quality and sample relationships

Dissection of the seven floral tissues generated 21 RNA-seq libraries, comprising three biological replicates per tissue (**Figure 1**). After quality filtering, the libraries yielded 302.83 Gb of clean data, with an average of approximately 48.12 million clean read pairs and 14.42 Gb per library. Q30 values ranged from 88.70% to 92.04%, and GC content ranged from 43.5% to 44.0% (**Supplementary Table 1**). Alignment to the *Camellia sinensis* cv. ‘Shuchazao’ v2 reference genome produced overall alignment rates of 87.34%–89.29% and a mean concordant paired-end alignment rate of 82.38% (**Supplementary Table 2**).

In the PCA, biological replicates from the same tissue clustered closely, whereas the seven tissues were clearly separated (**Figure 2A**). Pearson correlation coefficients among biological replicates of the same tissue were all greater than 0.92 (**Figure 2B**). These results supported the reproducibility of the RNA-seq data and their suitability for differential expression and co-expression analyses.

**Figure 2:**
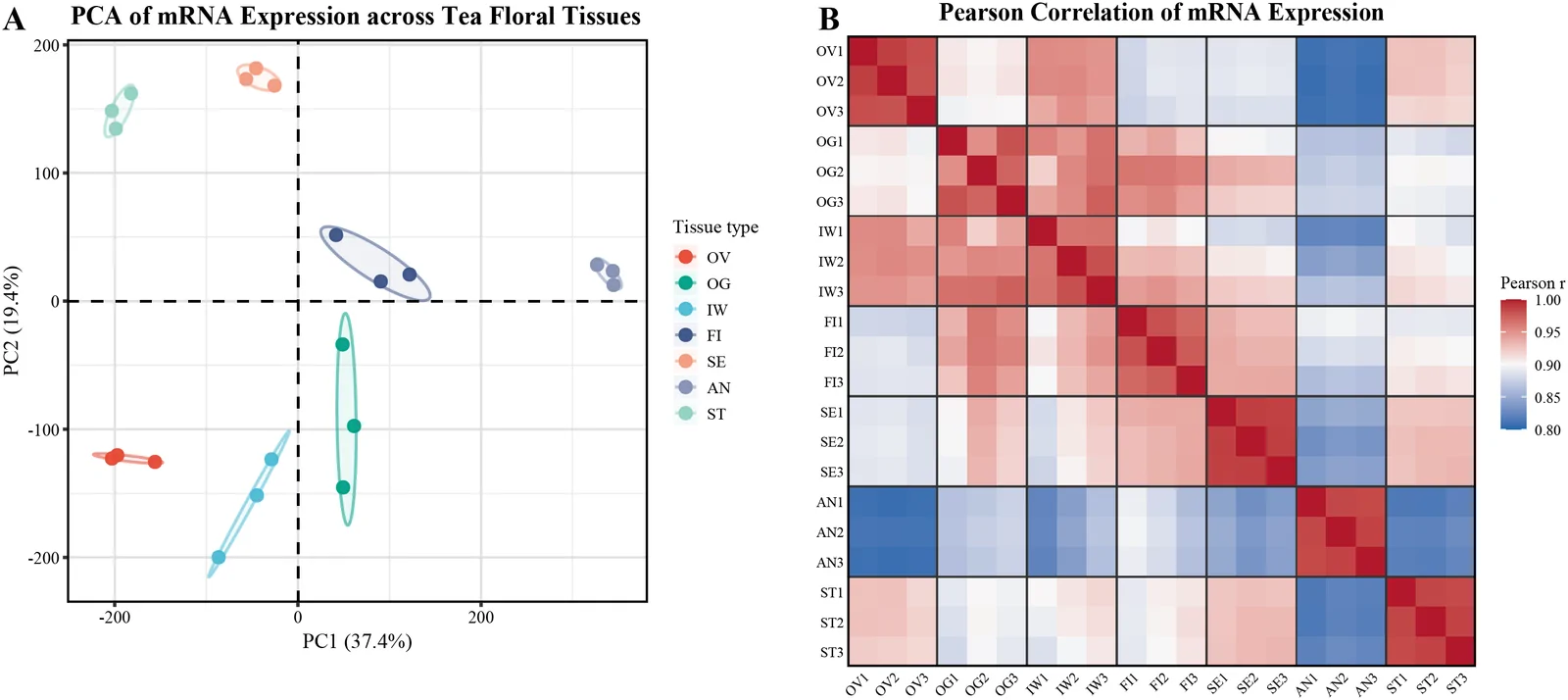
RNA-seq sample relationships among the seven tea floral tissues. **(A)** Principal component analysis based on log_2_(FPKM + 1)-transformed expression values of all quantified genes across the 21 RNA-seq libraries. Each point represents one biological replicate, and ellipses delineate samples from the same tissue. **(B)** Pearson correlation heatmap based on log2(FPKM + 1)-transformed gene expression values across the 21 RNA-seq libraries. OV, ovary; OG, outer green petal; IW, inner white petal; FI, filament; SE, sepal; AN, anther; ST, style.

### 3.2 Transcriptomic divergence among tea floral tissues

Pairwise differential expression analysis was performed for all 21 comparisons among the seven tissues. Using |log_2_ fold change| ≥ 1 and a Benjamini–Hochberg-adjusted *P* value < 0.05, 33,333 nonredundant DEGs were identified (**Figure 3A**). OG and IW showed the fewest DEGs, with 3,266 genes, whereas AN and ST showed the most, with 21,574 genes. Of the AN–ST DEGs, 11,898 showed higher expression in AN and 9,676 showed higher expression in ST. Euclidean distance analysis showed the same overall pattern: OG and IW had the smallest distance, approximately 155, whereas AN and ST had the largest, approximately 354 (**Figure 3B**).

**Figure 3:**
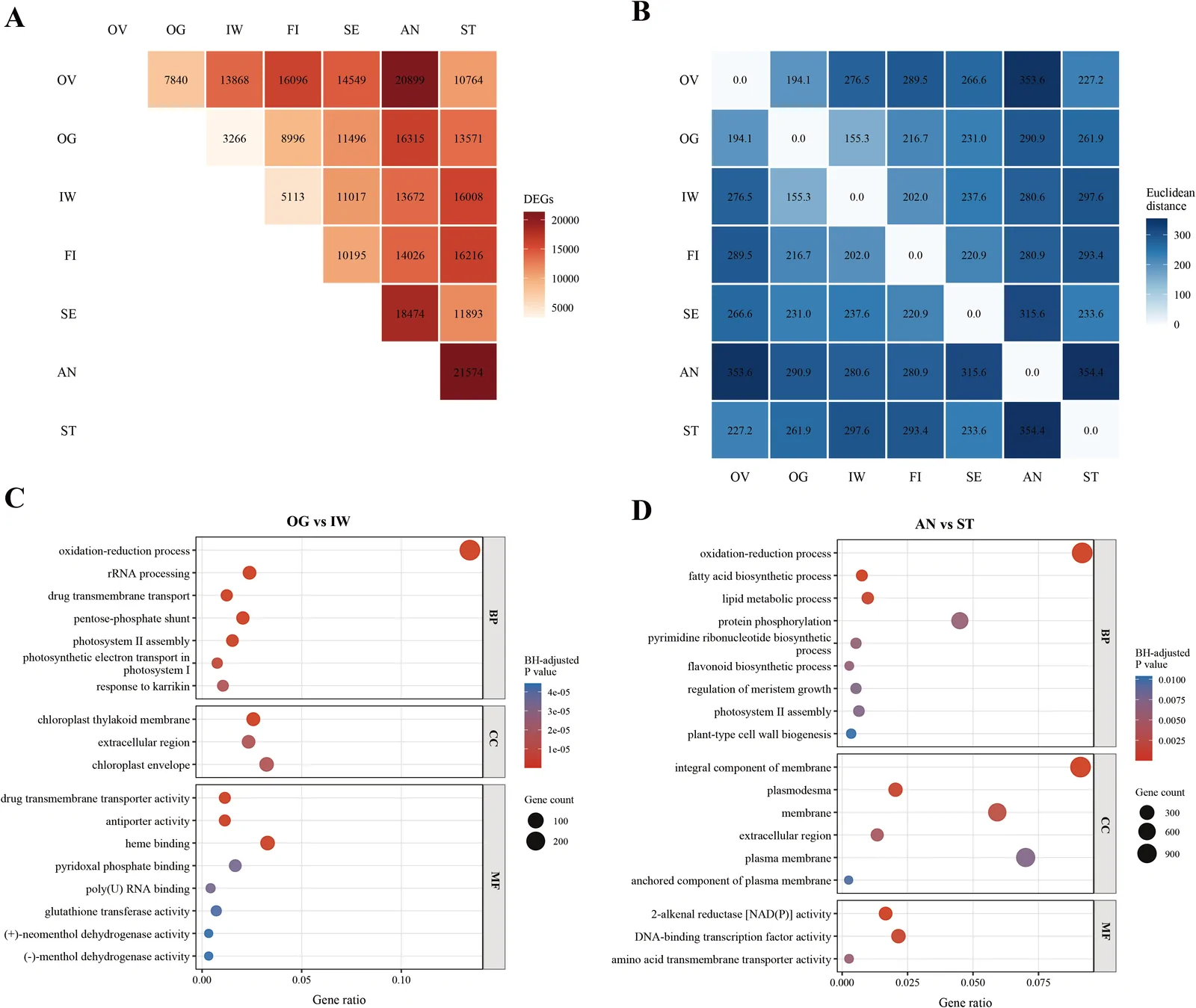
Transcriptomic divergence among the seven tea floral tissues. **(A)** Pairwise DEG count matrix for the 21 tissue comparisons. DEGs were defined using |log2 fold change| ≥ 1 and a Benjamini–Hochberg-adjusted *P* value < 0.05. Values in the cells indicate the total number of DEGs in each comparison. **(B)** Euclidean distance heatmap based on tissue-mean expression profiles. Raw counts were filtered, normalized using the trimmed mean of M values method, converted to counts per million, averaged across the three biological replicates within each tissue, and standardized across the seven tissues for each gene before distance calculation. **(C)** Selected significantly enriched GO terms among the DEGs between OG and IW, the comparison containing the fewest DEGs. **(D)** Selected significantly enriched GO terms among the DEGs between AN and ST, the comparison containing the most DEGs. Bubble size represents the number of genes assigned to each GO term, and color represents the Benjamini–Hochberg-adjusted *P* value. BP, biological process; CC, cellular component; MF, molecular function. OV, ovary; OG, outer green petal; IW, inner white petal; FI, filament; SE, sepal; AN, anther; ST, style.

DEGs between OG and IW were enriched in oxidation–reduction processes, rRNA processing, the pentose-phosphate shunt, photosystem II assembly, photosynthetic electron transport in photosystem I, chloroplast thylakoid membrane, and chloroplast envelope (**Figure 3C**). Thus, although OG and IW had similar global expression profiles, the genes that distinguished them were mainly associated with chloroplast structure, photosynthesis, and related metabolism. DEGs between AN and ST were enriched in oxidation–reduction processes, fatty acid biosynthesis, lipid metabolism, protein phosphorylation, flavonoid biosynthesis, regulation of meristem growth, plant-type cell wall biogenesis, plasmodesmata, and DNA-binding transcription factor activity (**Figure 3D**). The AN–ST contrast therefore encompassed broad differences in metabolic activity, cellular architecture, and signaling. RT-qPCR analysis of six representative genes showed tissue expression patterns broadly concordant with the RNA-seq estimates (**Figure 4**), supporting the reliability of the transcriptomic data.

**Figure 4:**
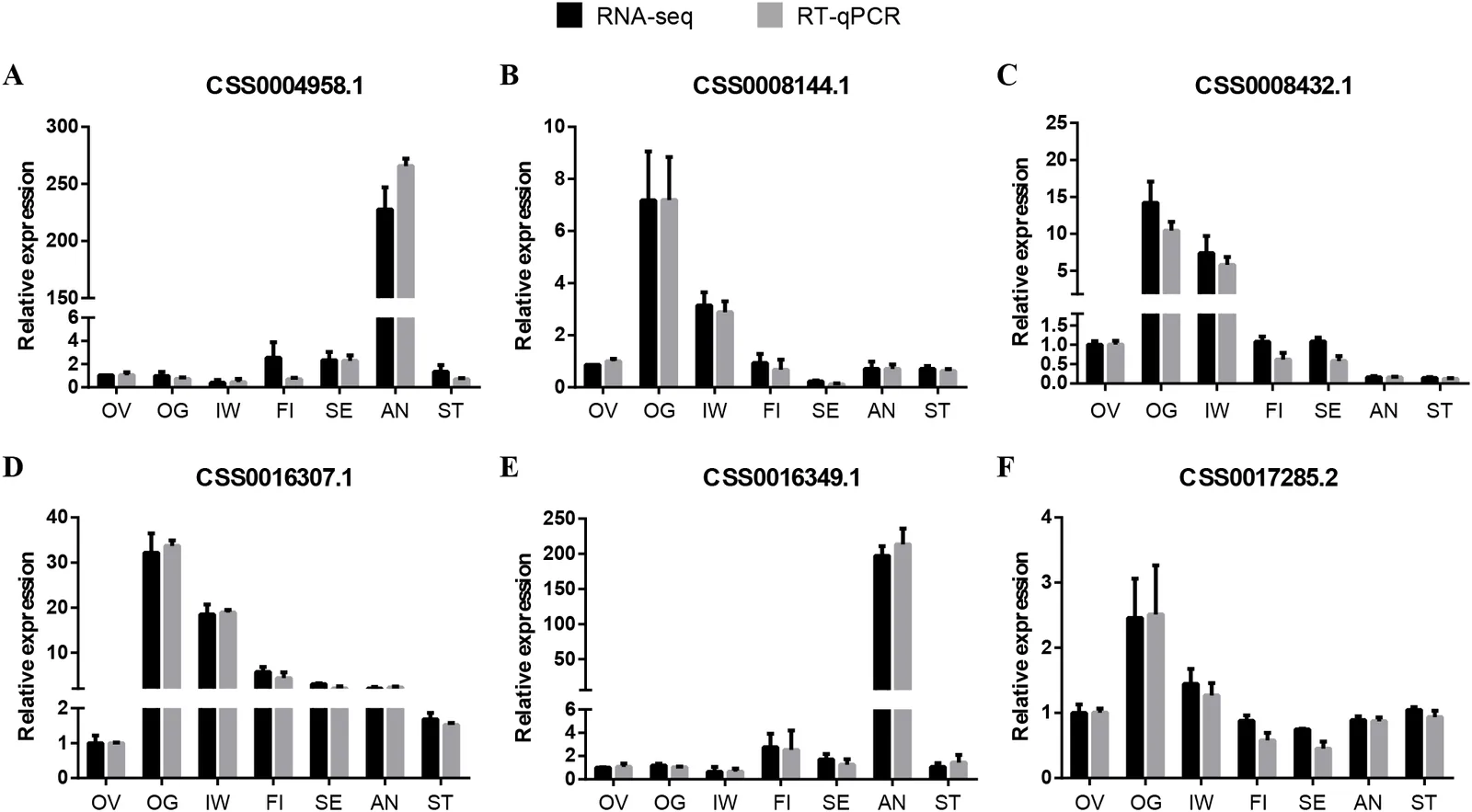
RT-qPCR validation of RNA-seq expression patterns. **(A–F)** Comparison of RNA-seq- and RT-qPCR-derived relative expression patterns for six representative genes across the seven tea floral tissues. Black bars represent RNA-seq-derived relative expression values, and gray bars represent RT-qPCR-derived relative expression values. For visual comparison, the expression values obtained using both methods were normalized relative to OV. RT-qPCR expression was calculated using the 2^−ΔΔCt^ method, with *β-actin* as the internal reference. Data are presented as mean ± SD of three biological replicates. Broken y-axes are used in **(A, C–E)** to display large differences in expression magnitude. OV, ovary; OG, outer green petal; IW, inner white petal; FI, filament; SE, sepal; AN, anther; ST, style.

### 3.3 Construction of co-expression networks and module–tissue trait relationships

A weighted gene co-expression network was constructed from the expression profiles of the 33,333 nonredundant DEGs across the 21 samples. A soft-thresholding power of 7 was selected because the scale-free topology fit index exceeded 0.80 at this value (**Supplementary Figure 1**). Dynamic tree cutting followed by module merging identified 24 co-expression modules (**Supplementary Figure 2**). Module–trait correlation analysis identified seven modules whose eigengenes showed positive correlations with the corresponding binary tissue traits (**Figure 5A**; **Supplementary Table 4**). The white module eigengene correlated positively with the OV trait (*r* = 0.92, *P* = 6 × 10^−9^) and was enriched in chloroplast-related structures, photosystem assembly, and the pentose phosphate pathway. The bisque4 module eigengene correlated positively with the IW trait (*r* = 0.82, *P* = 6 × 10^−6^) and was enriched in the extracellular region, fatty acid biosynthesis, and peroxidase activity. The cyan module eigengene correlated positively with the OG trait (*r* = 0.72, *P* = 3 × 10^−4^) and was enriched in monooxygenase activity, oxidation–reduction processes, and metal ion binding. The magenta module eigengene correlated positively with the FI trait (*r* = 0.99, *P* = 5 × 10^−16^) and was enriched in lipid metabolism, carotenoid biosynthesis, and intracellular transport. The red module eigengene correlated positively with the SE trait (*r* = 0.98, *P* = 4 × 10^−15^) and was enriched in plant-type cell wall, cell wall organization, polysaccharide biosynthesis, and carbohydrate metabolism. The turquoise module eigengene correlated positively with the AN trait (*r* = 0.99, *P* = 5 × 10^−22^) and was enriched in pollen tube tip, pollen tube growth, actin binding, calcium ion binding, and vesicle-mediated transport. The yellow module eigengene correlated positively with the ST trait (*r* = 0.83, *P* = 4 × 10^−6^) and was enriched in RNA methylation, translation, DNA replication, nucleosome assembly, and chromatin regulation. These modules showed distinct tissue-associated eigengene patterns and functional enrichment profiles.

**Figure 5:**
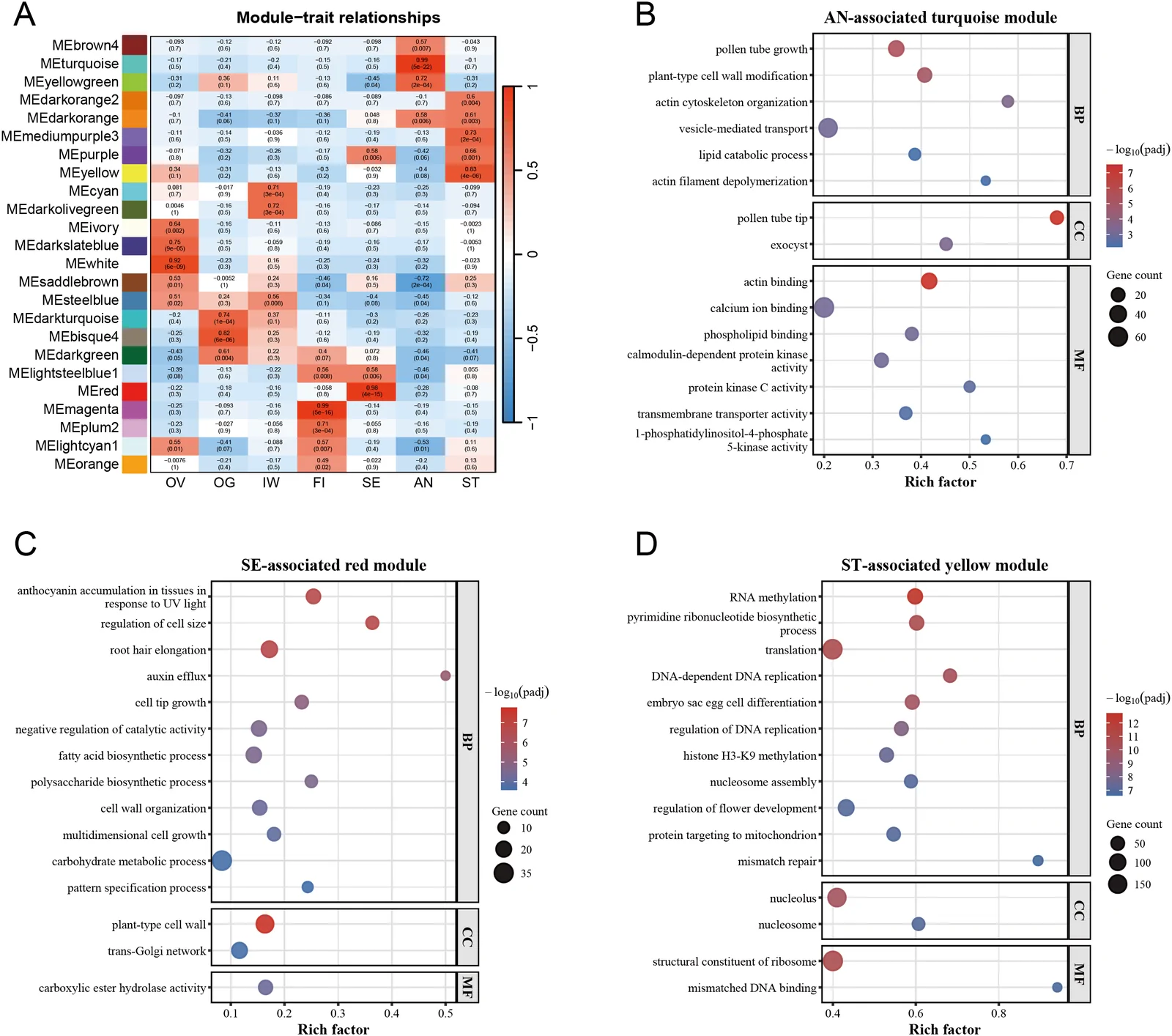
Module–tissue trait relationships and functional enrichment of candidate hub genes in three representative co-expression modules. **(A)** Module eigengene–tissue trait correlation heatmap showing Pearson correlations between module eigengenes and binary tissue traits. Values in the cells indicate correlation coefficients, with corresponding *P* values shown in parentheses. **(B–D)** GO enrichment bubble plots for candidate hub genes in the AN-associated turquoise module, SE-associated red module, and ST-associated yellow module, respectively. The x-axis represents the rich factor. Bubble size represents gene count, and color represents −log10 of the Benjamini–Hochberg-adjusted *P* value. BP, biological process; CC, cellular component; MF, molecular function. OV, ovary; OG, outer green petal; IW, inner white petal; FI, filament; SE, sepal; AN, anther; ST, style.

### 3.4 Candidate hub gene identification and co-expression network analysis of three representative modules

A total of 111 candidate hub genes were selected from the AN-associated turquoise module, the SE-associated red module, and the ST-associated yellow module. The three modules contained 38, 35, and 38 candidate hub genes, respectively (**Figures 5B–D**, **6A–C**; **Supplementary Table 4**).

**Figure 6:**
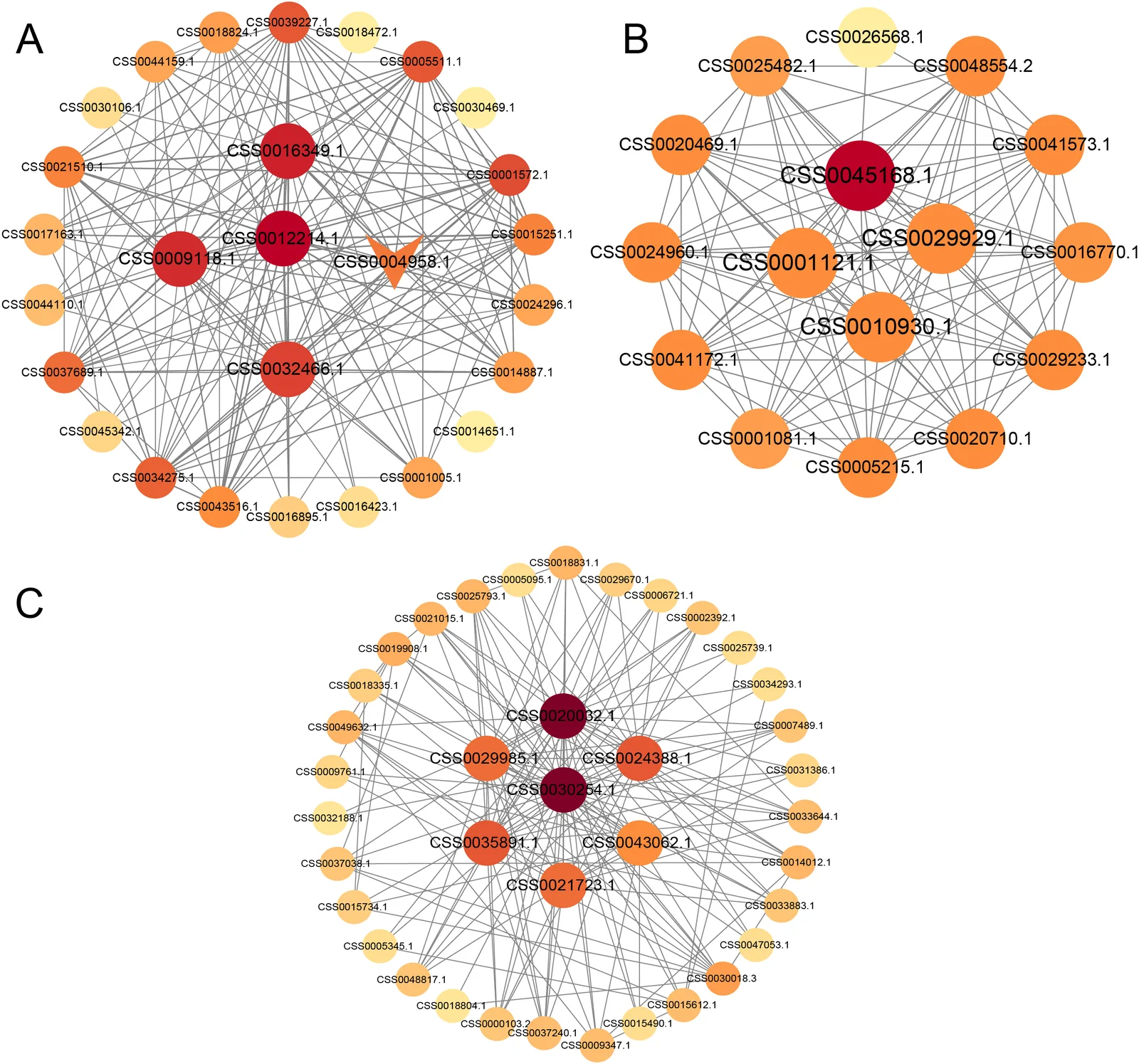
Co-expression networks of candidate hub genes in three representative modules. **(A)** Co-expression network of candidate hub genes in the AN-associated turquoise module. **(B)** Co-expression network of candidate hub genes in the SE-associated red module. **(C)** Co-expression network of candidate hub genes in the ST-associated yellow module. Nodes represent candidate hub genes, and gray edges represent TOM-derived mRNA–mRNA co-expression relationships retained for network visualization. Node color indicates degree within the displayed network, with darker colors representing higher degree. A subset of the highest-degree nodes in each network was enlarged and labeled to facilitate visualization. In **(A)**, the inverted triangle represents CSS0004958.1, one of the genes examined by RT-qPCR; all other candidate hub genes are represented by circles. TOM weight thresholds of 0.70, 0.47, and 0.50 were applied to the turquoise, red, and yellow module networks, respectively.

Candidate hub genes in the turquoise module were enriched in pollen germination, pollen tube growth, actin cytoskeleton organization, Ca^2+^-related signaling, vesicle-mediated transport, and cell wall modification (**Figures 5B**, **6A**). This functional profile is consistent with processes required for pollen maturation, germination, and subsequent polarized growth. Candidate hub genes in the red module were enriched in cell wall organization, pectin modification, polysaccharide biosynthesis, carbohydrate metabolism, extracellular structures, and lipid-related processes (**Figures 5C**, **6B**), consistent with cell wall formation and tissue expansion in sepals. Candidate hub genes in the yellow module were enriched in RNA processing, translation, DNA replication, nucleosome assembly, chromatin organization, DNA repair, and floral development-related transcriptional regulation (**Figures 5D**, **6C**), indicating prominent nucleic acid metabolic and developmental regulatory activities in the style. These three networks provided the tissue-associated mRNA contexts used for subsequent integration with predicted miRNA–mRNA relationships.

### 3.5 Identification and expression profiling of miRNAs in seven floral tissues

Small RNA sequencing of the same 21 biological samples yielded 333,195,098 clean reads after adapter trimming and quality filtering, corresponding to 11,047,334–26,494,432 reads per library and 8.815 Gb of sequence data. GC content ranged from 45% to 48%, and no sequences were flagged as poor quality. All libraries passed the FastQC modules for per-base sequence quality, per-sequence quality scores, per-base N content, and adapter content. Alignment rates to the *Camellia sinensis* cv. ‘Shuchazao’ v2 reference genome exceeded 85.63% for all libraries, with a mean of 88.85% (**Supplementary Table 5**). Biological replicates from the same tissue generally clustered together in the PCA, whereas the seven tissues were separated (**Figure 7A**). Pearson correlation coefficients between biological replicates of the same tissue were all greater than 0.88 (**Figure 7B**).

**Figure 7:**
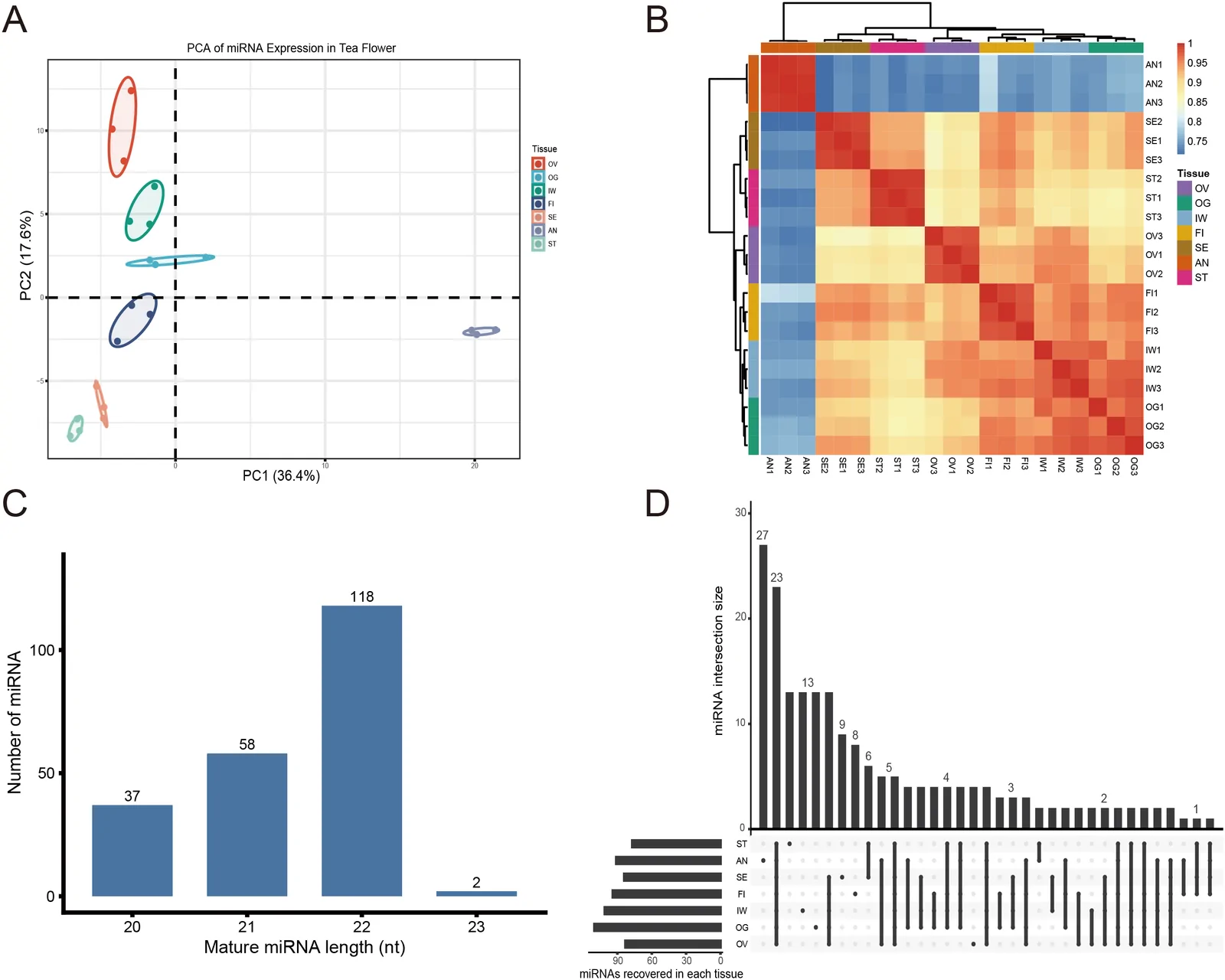
Identification and tissue-level recovery of miRNAs in tea floral tissues. **(A)** Principal component analysis based on variance-stabilized miRNA count data across the 21 small RNA-seq libraries. Each point represents one biological replicate, and ellipses delineate samples from the same tissue. **(B)** Hierarchically clustered Pearson correlation heatmap based on variance-stabilized miRNA count data. **(C)** Length distribution of the 215 representative mature miRNA sequences. **(D)** UpSet plot showing the tissue-level recovery and intersections of the identified miRNA loci. A miRNA locus was considered recovered in a tissue when it was predicted by miRDP2 in at least one of the three libraries from that tissue. Horizontal bars indicate the number of miRNA loci recovered from each tissue, vertical bars indicate intersection sizes, and connected dots indicate the tissues included in each intersection. OV, ovary; OG, outer green petal; IW, inner white petal; FI, filament; SE, sepal; AN, anther; ST, style.

A total of 215 nonredundant miRNAs were identified after miRDP2 prediction, Rfam filtering, and miRBase release 22 homology analysis. Of these, 54 were homologs of known plant miRNA families representing 14 families, and 161 were classified as putative novel miRNAs (**Supplementary Figure 3**; **Supplementary Table 6**). Representative mature miRNA sequences ranged from 20 to 23 nt. The 22-nt class was predominant, containing 118 miRNAs, followed by the 21-, 20-, and 23-nt classes, which contained 58, 37, and 2 miRNAs, respectively (**Figure 7C**). Eighty-four miRNAs had U as their 5′ terminal nucleotide, accounting for 39.1% of the identified miRNAs, and the 20–22-nt classes showed a clear 5′ U preference (**Supplementary Figure 4**). UpSet analysis showed that 27 miRNA loci were recovered only from AN libraries, representing the largest single-tissue intersection, whereas 23 loci were recovered from all seven tissues. Additional single-tissue intersections comprised 13 miRNA loci for each of OG, IW, and ST (**Figure 7D**).

Across the 21 pairwise tissue comparisons, 175 nonredundant differentially expressed miRNAs were identified. The OV–AN comparison contained the largest number of differentially expressed miRNAs, with 107 in total, including 42 with higher expression in OV and 65 with higher expression in AN. The OG–IW comparison contained the fewest differentially expressed miRNAs, with 21 in total, including 16 with higher expression in OG and five with higher expression in IW. In all six comparisons involving AN, more miRNAs showed higher expression in AN than in the corresponding comparison tissue.

### 3.6 Integrated analysis of miRNAs and candidate hub genes in tissue-associated modules

psRNATarget predicted 25,835 nonredundant miRNA–mRNA relationships involving 215 miRNAs and 12,196 candidate target genes. Intersecting the predicted targets with candidate hub genes from the AN-associated turquoise, SE-associated red, and ST-associated yellow modules yielded 45 predicted miRNA–candidate hub gene relationships involving 39 miRNAs and 28 candidate hub genes (**Figures 8A–C**; **Supplementary Table 6**).

**Figure 8:**
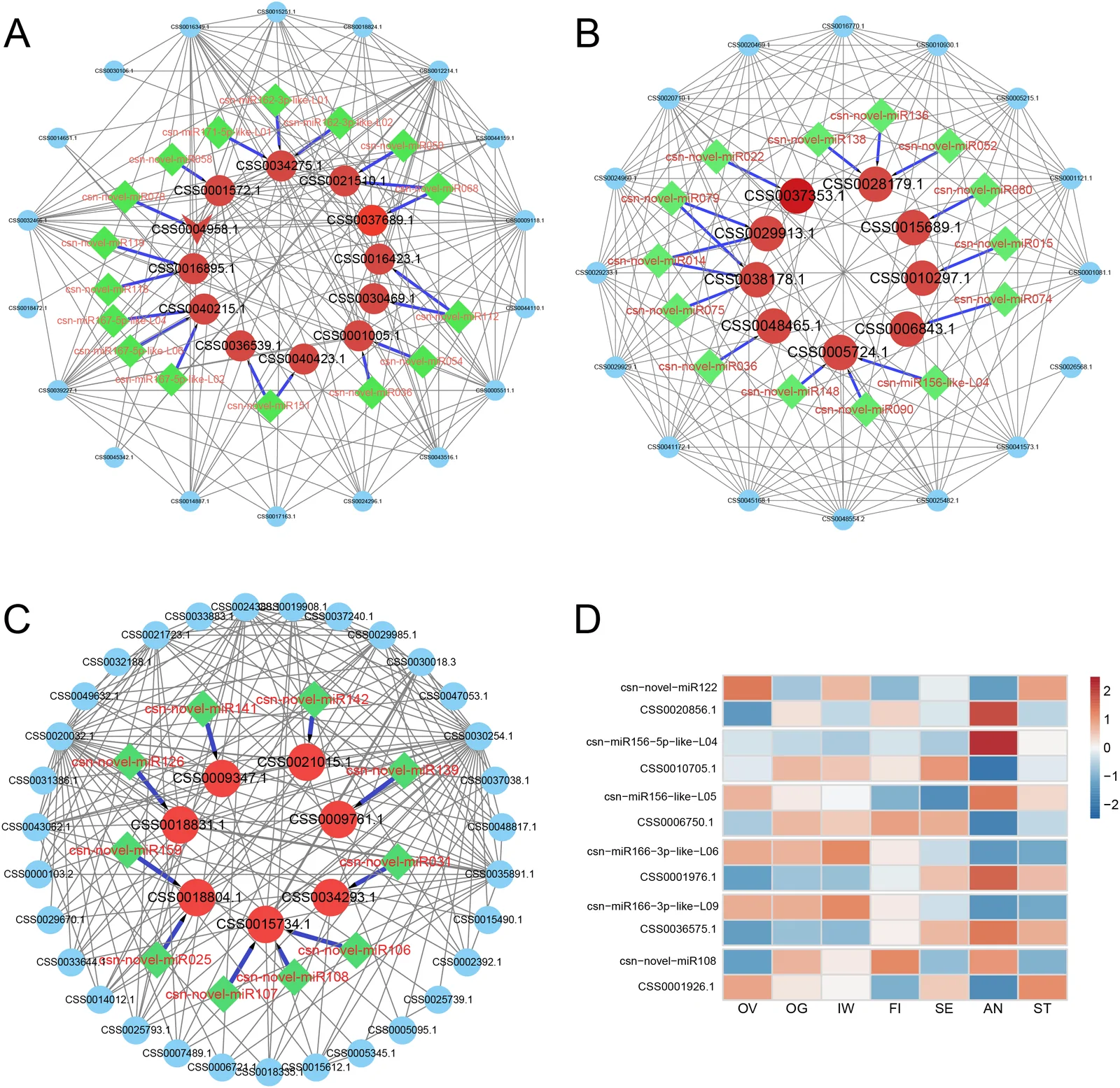
Integrated miRNA–mRNA networks and expression patterns of six representative predicted miRNA–mRNA pairs showing negative correlations. **(A–C)** Integrated networks constructed by adding psRNATarget-predicted miRNA–mRNA relationships to the TOM-derived mRNA co-expression networks of candidate hub genes. **(A)** AN-associated turquoise module network. **(B)** SE-associated red module network. **(C)** ST-associated yellow module network. Green diamond-shaped nodes represent miRNAs, red circular nodes represent candidate hub genes targeted by predicted miRNAs, and blue circular nodes represent other candidate hub genes retained in the corresponding co-expression networks. Gray edges represent TOM-derived mRNA–mRNA co-expression relationships, whereas blue directed edges represent psRNATarget-predicted miRNA–mRNA relationships. **(D)** Heatmap of tissue-mean expression patterns for six representative predicted miRNA–mRNA pairs showing significant negative Spearman correlations across the 21 matched samples. Expression values were standardized separately for each miRNA or mRNA and are displayed as row Z-scores. The color scale indicates the row Z-score. OV, ovary; OG, outer green petal; IW, inner white petal; FI, filament; SE, sepal; AN, anther; ST, style.

The predicted miRNA–mRNA relationships were added to the corresponding mRNA co-expression networks shown in **Figures 6A–C**. The AN-associated turquoise network comprised 19 predicted relationships between 16 miRNAs and 12 candidate hub genes. The 12 candidate hub genes were associated mainly with pollen tube growth, actin cytoskeleton organization, Ca^2+^-related signaling, vesicle-mediated transport, and cell wall modification. The SE-associated red network comprised 16 predicted relationships between 14 miRNAs and nine candidate hub genes associated with cell wall and pectin remodeling, polysaccharide metabolism, and auxin transport. The ST-associated yellow network comprised ten predicted relationships between ten miRNAs and seven candidate hub genes associated with RNA processing, translation, chromatin regulation, and floral development-related transcriptional regulation. In all three networks, individual candidate hub genes were predicted to be targeted by multiple miRNAs. In the turquoise and red networks, some miRNAs were also predicted to target multiple candidate hub genes.

Spearman correlation analysis was performed for all psRNATarget-predicted relationships across the 21 matched samples. Six representative pairs involving distinct miRNAs and mRNAs were selected from those showing significant negative correlations. Their Spearman’s *ρ* values ranged from −0.953 to −0.847, and all BH-adjusted *P* values were below 0.05. At the tissue-mean level, the miRNAs and their corresponding predicted target genes generally showed inverse relative expression patterns across the seven tissues (**Figure 8D**; **Supplementary Table 7**).

## 4 Discussion

### 4.1 Transcriptomic differentiation among tea floral tissues

The separation of the seven tissues in the PCA and the large number of DEGs indicate substantial transcriptional specialization within tea flower buds at the bud-white stage. This pattern is consistent with the different functions of protective outer organs and male and female reproductive tissues. Previous tea flower transcriptomic studies mainly examined mixtures of floral organs, successive developmental stages, hormone-associated developmental processes, or abnormal reproductive phenotypes (Tan et al., 2013; Liu et al., 2017, 2020b; Xu et al., 2022). Organ-resolved analyses in *Camellia sasanqua* and *Cymbidium ensifolium* also revealed pronounced expression differences among floral tissues (Yang et al., 2021; Fan et al., 2022). By profiling dissected tissues separately, the present study resolved transcriptional differences that may be masked when whole flowers or mixed floral organs are analyzed.

OG and IW had the fewest DEGs and the smallest Euclidean distance, consistent with their shared petal identity and related anatomical features. The DEGs separating these tissues were enriched in oxidation–reduction processes, rRNA processing, the pentose-phosphate shunt, photosystem II assembly, photosynthetic electron transport in photosystem I, and chloroplast-associated compartments. Studies of green and non-green chrysanthemum petals have linked petal greening to chlorophyll metabolism, chloroplast maintenance, and photosynthesis-related gene expression (Ohmiya et al., 2017; Fu et al., 2021). In *Arabidopsis*, CIN-like TCP factors restrict petal greening by repressing chlorophyll biosynthesis and promoting plastid differentiation (Zheng et al., 2022). The enrichment of chloroplast- and photosynthesis-related terms in the OG–IW comparison suggests that plastid-associated processes are a major source of molecular differentiation between the two petal tissues, despite their overall transcriptomic similarity.

AN and ST had the largest number of DEGs and the greatest Euclidean distance, consistent with their distinct cellular composition and reproductive functions. Anthers support microsporogenesis, pollen maturation, and pollen release, whereas the style forms part of the female reproductive tract and provides the extracellular environment through which pollen tubes grow toward the ovary (Broz and Bedinger, 2021; Zhong et al., 2024). AN–ST DEGs were enriched in oxidation–reduction processes, fatty acid and lipid metabolism, protein phosphorylation, flavonoid biosynthesis, regulation of meristem growth, plant-type cell wall biogenesis, plasmodesmata, and DNA-binding transcription factor activity. These enrichments indicate extensive differentiation of metabolic, structural, and signaling programs between the male and female reproductive tissues. The importance of flavonoid metabolism in tea anthers is further supported by functional analyses of tea flavonol synthase genes involved in anther development (Shi et al., 2021). The tissue-resolved comparisons distinguish shared petal programs from expression programs associated with male and female reproductive tissues. These differences may be overlooked in whole-flower or mixed-organ transcriptomes. The analysis also links individual floral tissues with specific biological processes and identifies candidate pathways and genes for further functional studies of floral organ differentiation and reproductive development in tea.

### 4.2 The AN-associated module is enriched for processes related to pollen germination and polarized growth

The eigengene of the turquoise module showed a strong positive correlation with the AN trait, and the module was enriched in pollen germination, pollen tube growth, actin cytoskeleton organization, calcium ion binding, vesicle-mediated transport, and plant-type cell wall modification. Pollen tube growth is a highly polarized process requiring coordinated cytoskeletal dynamics, intracellular transport, membrane trafficking, and cell wall remodeling (Hepler et al., 2013; Xu and Huang, 2020; Duckney et al., 2021; Zhong et al., 2024). Because the sampled anthers had not dehisced and pollen grains remained within the anther locules, the detected expression program is consistent with a pre-dehiscence state that supports pollen maturation and prepares pollen for germination and subsequent tube growth.

Actin organization and Ca^2+^ signaling are central to polarized pollen tube growth. Longitudinal actin bundles support cytoplasmic streaming and vesicle transport, whereas dynamic actin structures near the apex regulate vesicle distribution and growth direction (Hepler et al., 2013; Xu and Huang, 2020; Duckney et al., 2021). Disruption of NET2-mediated connections between F-actin and the plasma membrane alters cortical actin organization and pollen tube polarity (Duckney et al., 2021). Within the turquoise network, CSS0040215.1 was associated with GO terms related to actin binding and actin cytoskeleton organization, whereas CSS0016423.1 and CSS0030469.1 were associated with calcium ion binding and calmodulin-dependent protein kinase activity. The apical Ca^2+^ gradient regulates actin turnover, secretion, and directional growth (Cárdenas et al., 2008). CSS0034275.1 was associated with SNAP receptor activity, membrane fusion, and vesicle-mediated transport, linking vesicle trafficking to cytoskeletal and Ca^2+^-related processes within the AN-associated network. Rho GTPase-dependent actin dynamics similarly coordinate vesicle targeting and exocytosis during tip growth (Lee et al., 2008).

Spatial regulation of the pollen tube wall permits rapid apical extension while maintaining mechanical integrity. The apex contains relatively extensible methylesterified pectin, whereas de-esterified pectin and callose accumulate behind the tip and strengthen the wall (Bosch et al., 2005; Hepler et al., 2013). CSS0021510.1 was associated with callose synthesis, pollen exine formation, pollen germination, and pollen tube growth, whereas CSS0001572.1 was associated with pectinesterase activity and pectin modification. Functional analysis of *PbrBGAL6* in pear showed that changes in apical pectin composition affect pollen tube length, tip morphology, and wall integrity (Xu et al., 2025). The co-enrichment of cytoskeletal organization, Ca^2+^ signaling, vesicle trafficking, and wall modification therefore supports a coordinated pollen-related program in the AN-associated module.

The AN integrated network contained 19 predicted relationships involving 16 miRNAs and 12 candidate hub genes. Multiple miR162-like and miR171-like members were predicted to target the vesicle-transport-related gene CSS0034275.1, whereas several miR167-5p-like members were predicted to target the actin-related gene CSS0040215.1. csn-novel-miR112 was predicted to target both CSS0016423.1 and CSS0030469.1, two candidates associated with Ca^2+^ signaling and protein kinase activity. These predictions identify a focused set of miRNA–mRNA pairs associated with several components of the AN-associated expression program for subsequent experimental analysis, consistent with integrative studies of floral miRNA regulation in other species (Zheng et al., 2024).

### 4.3 The SE-associated module reflects cell wall remodeling and growth regulation

The eigengene of the red module showed a strong positive correlation with the SE trait, and the module was enriched in plant-type cell wall, cell wall organization, polysaccharide biosynthesis, and carbohydrate metabolism. Sepals form the outermost floral whorl and expand while enclosing the developing inner organs. Studies of *Arabidopsis* sepals have shown that reproducible organ size and shape depend on coordinated cellular growth, cell wall mechanics, and mechanical feedback (Hervieux et al., 2016; Roeder, 2021). The functional profile of the red module is therefore consistent with the structural and growth requirements of developing tea sepals.

Candidate hub genes in the red module were associated with pectin modification, cellulose metabolism, cell wall biogenesis, and cell growth. Pectin methylesterification alters wall mechanical properties and can promote organ outgrowth (Peaucelle et al., 2011), whereas cellulose organization contributes to anisotropic sepal expansion (Hervieux et al., 2016). The module also contained an auxin efflux transporter and a candidate gene associated with auxin signaling and cell size regulation. In tomato and spinach, auxin biosynthesis and transport contribute to sepal expansion, morphology, and persistence (Li et al., 2017; Fatima et al., 2021). The SE-associated program may therefore integrate cell wall composition, mechanical properties, and hormone-dependent growth.

The SE integrated network contained 16 predicted relationships involving 14 miRNAs and nine candidate hub genes. The predicted targets were associated mainly with pectin remodeling, polysaccharide metabolism, cell wall biogenesis, and auxin transport. The integrated analysis therefore prioritizes candidate miRNA–mRNA relationships within an mRNA program related to sepal structure and expansion.

### 4.4 The ST-associated module is enriched for RNA metabolism and chromatin-related developmental processes

The eigengene of the yellow module showed a positive correlation with the ST trait, and the module was enriched in RNA processing, RNA methylation, translation, DNA replication, nucleosome assembly, chromatin organization, and developmental transcriptional regulation. The style is a central component of the female reproductive tract and contains the transmitting tract through which pollen tubes grow from the stigma toward the ovary. Transmitting tract cells produce an extracellular matrix that supports pollen tube adhesion, nutrition, growth, and guidance (Pereira et al., 2021). Transcriptomic analyses of tobacco stigmas and styles and *Arabidopsis* pistils identified preferential expression of genes involved in secretion, extracellular matrix remodeling, signaling, and pollen–pistil interactions (Tung et al., 2005; Quiapim et al., 2009). The RNA-processing and translation-related functions enriched in the ST module may therefore reflect the biosynthetic and regulatory activity required for style maturation and establishment of a functional transmitting tract.

Chromatin regulation may also contribute to the establishment and maintenance of style developmental programs. In *Arabidopsis*, NGATHA factors are required for style specification and interact with auxin-dependent developmental pathways (Trigueros et al., 2009). HISTONE DEACETYLASE19 regulates transmitting tract differentiation through histone-acetylation-dependent control of *SHOOT MERISTEMLESS* (Manrique et al., 2024), whereas the trithorax-group regulator ULTRAPETALA1 interacts with KANADI factors during gynoecium patterning (Pires et al., 2014). These studies provide a biological context for the enrichment of nucleosome assembly, histone modification, and chromatin organization in the tea ST module.

The ST integrated network comprised ten predicted miRNA–mRNA relationships involving ten miRNAs and seven candidate hub genes. CSS0015734.1, which was associated with RNA processing and transcriptional regulation, was predicted to be targeted by csn-novel-miR106, csn-novel-miR107, and csn-novel-miR108. Other predicted targets were associated with translation, RNA binding, histone modification, and floral development. These predictions place candidate miRNA–mRNA relationships within the RNA-metabolic and chromatin-associated expression program of the style.

### 4.5 Tissue-dependent miRNA expression and candidate miRNA–mRNA relationships

The identified mature miRNAs ranged from 20 to 23 nt, with 22-nt miRNAs forming the largest class. Plant miRNAs are commonly 21 or 22 nt long, and both length and the 5′ terminal nucleotide influence ARGONAUTE loading and downstream silencing functions (Mi et al., 2008; Chen et al., 2010; Cuperus et al., 2010). The observed 5′ U preference is consistent with the sequence bias of AGO1-associated plant miRNAs (Mi et al., 2008). Because annotation of novel plant miRNAs from sequencing data alone is prone to false positives, sequences without qualifying miRBase matches were described conservatively as putative novel miRNAs (Axtell and Meyers, 2018).

miRNA expression differed markedly among the seven floral tissues. OV and AN showed the largest number of differentially expressed miRNAs, whereas OG and IW showed the fewest, paralleling the relative transcriptomic divergence observed in the mRNA data. In all six comparisons involving AN, more miRNAs had higher expression in AN than in the corresponding comparison tissue. Previous analysis of fertile and sterile tea flower buds identified differential expression of conserved families including miR156, miR164, miR167, miR172, and miR319 (Qu et al., 2021). The present study extends these flower-level observations by resolving differences in miRNA expression among individual tissues of normal tea flower buds. The UpSet analysis further identified miRNA loci recovered across all seven tissues and loci supported only by libraries from individual tissues. These patterns indicate differences in the tissue-level representation of miRNA candidates but do not establish strict tissue specificity without additional expression-based and experimental evidence.

Intersecting psRNATarget predictions with candidate hub genes from the turquoise, red, and yellow modules reduced the initial prediction set to 45 relationships involving 39 miRNAs and 28 candidate hub genes. This integration placed predicted targets within co-expression programs associated with AN, SE, and ST functions (Dai et al., 2018; Zhang et al., 2020). In the resulting networks, several candidate hub genes were predicted to be targeted by multiple miRNAs, and some miRNAs were predicted to target more than one hub gene.

Six representative predicted pairs showed significant negative correlations across the 21 matched samples. Two miR156-like members were paired with genes associated with lipid and fatty acid metabolism, whereas two miR166-3p-like members were paired with genes associated with β-galactosidase activity and carbohydrate metabolism. miR156 is best characterized through its regulation of *SPL* genes, which encode SQUAMOSA PROMOTER BINDING PROTEIN-LIKE transcription factors involved in developmental phase transitions (Wu et al., 2009), whereas the miR166–HD-ZIP III pathway contributes to reproductive development in tomato (Clepet et al., 2021). The tea targets predicted here differ from these canonical targets and therefore represent additional candidate associations of miR156-like and miR166-like members. The csn-novel-miR122–CSS0020856.1 and csn-novel-miR108–CSS0001926.1 pairs further represent candidate associations between putative novel miRNAs and genes related to phosphorylation and RNA processing. Their sequence complementarity and inverse expression patterns support prioritizing these pairs for experimental validation.

### 4.6 Biological significance, limitations, and future perspectives

Because RNA-seq and small RNA-seq libraries were generated from the same total RNA preparations, the 21 mRNA and miRNA profiles were directly matched. This design allowed tissue-dependent miRNA expression to be evaluated together with differential mRNA expression and tissue-associated co-expression modules. Integration with psRNATarget predictions further narrowed the candidate miRNA–mRNA relationships to those embedded in biologically interpretable mRNA networks (Dai et al., 2018; Zhang et al., 2020; Mo et al., 2022).

Several limitations should be considered. First, the study examined only the bud-white stage; therefore, the identified programs do not represent earlier or later stages of floral development. Second, each dissected tissue contained multiple cell types, and the bulk expression profiles represent tissue-level averages. Third, the putative novel miRNAs and predicted miRNA–mRNA relationships require experimental validation. Negative correlations across tissues were used only to prioritize representative pairs because such patterns can arise from both post-transcriptional regulation and differences in tissue composition. Single-cell or spatial transcriptomic approaches could localize the candidate programs to specific cell populations (Lee et al., 2025), whereas degradome sequencing, cleavage assays, reporter analyses, and genetic experiments could test selected miRNA–mRNA pairs. Finally, the use of the ‘Shuchazao’ reference genome for ‘Fudingdabaicha’ samples may affect read alignment, transcript quantification, and candidate-gene annotation because sequence and structural variants occur among tea cultivars (Chen et al., 2023).

This study characterizes paired mRNA and miRNA expression profiles across seven tea floral tissues and identifies tissue-associated co-expression programs related to anther, sepal, and style functions. Integration of small RNA profiles, target prediction, and mRNA co-expression networks further prioritizes candidate genes and miRNA–mRNA pairs for functional analysis of tea floral tissue differentiation and reproductive development.

## Data Availability

The datasets presented in this study can be found in online repositories. The names of the repository/repositories and accession number(s) can be found in the article/**Supplementary Material**.
