# Supplementary material for: Integrated RNA-seq and small RNA-seq profiling reveals tissue-associated transcriptional programs and candidate miRNA–mRNA relationships across tea floral tissues
Source: Front Plant Sci. 2026 Sep 14;17:1941110. doi: 10.3389/fpls.2026.1941110 (PMC13617024; doi:10.3389/fpls.2026.1941110)
Supplement: Supplementary file 1 [file Image1.pdf]

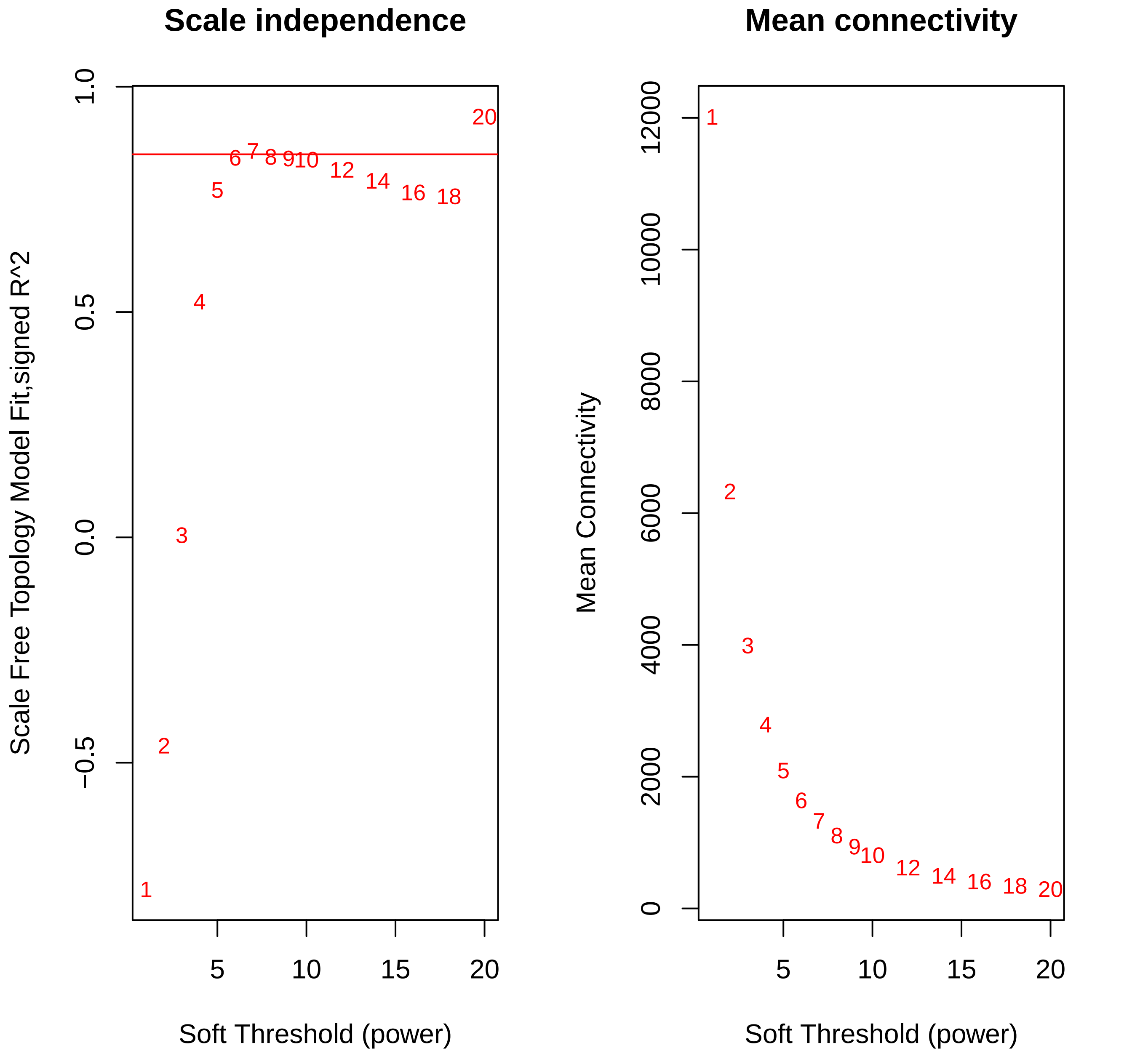
Scale independence
Mean connectivity
Scale Free Topology Model Fit,signed R^2
Soft Threshold (power)
Mean Connectivity
Soft Threshold (power)
