# Supplementary material for: Integrated RNA-seq and small RNA-seq profiling reveals tissue-associated transcriptional programs and candidate miRNA–mRNA relationships across tea floral tissues
Source: Front Plant Sci. 2026 Sep 14;17:1941110. doi: 10.3389/fpls.2026.1941110 (PMC13617024; doi:10.3389/fpls.2026.1941110)
Supplement: Supplementary file 2 [file Image2.pdf]

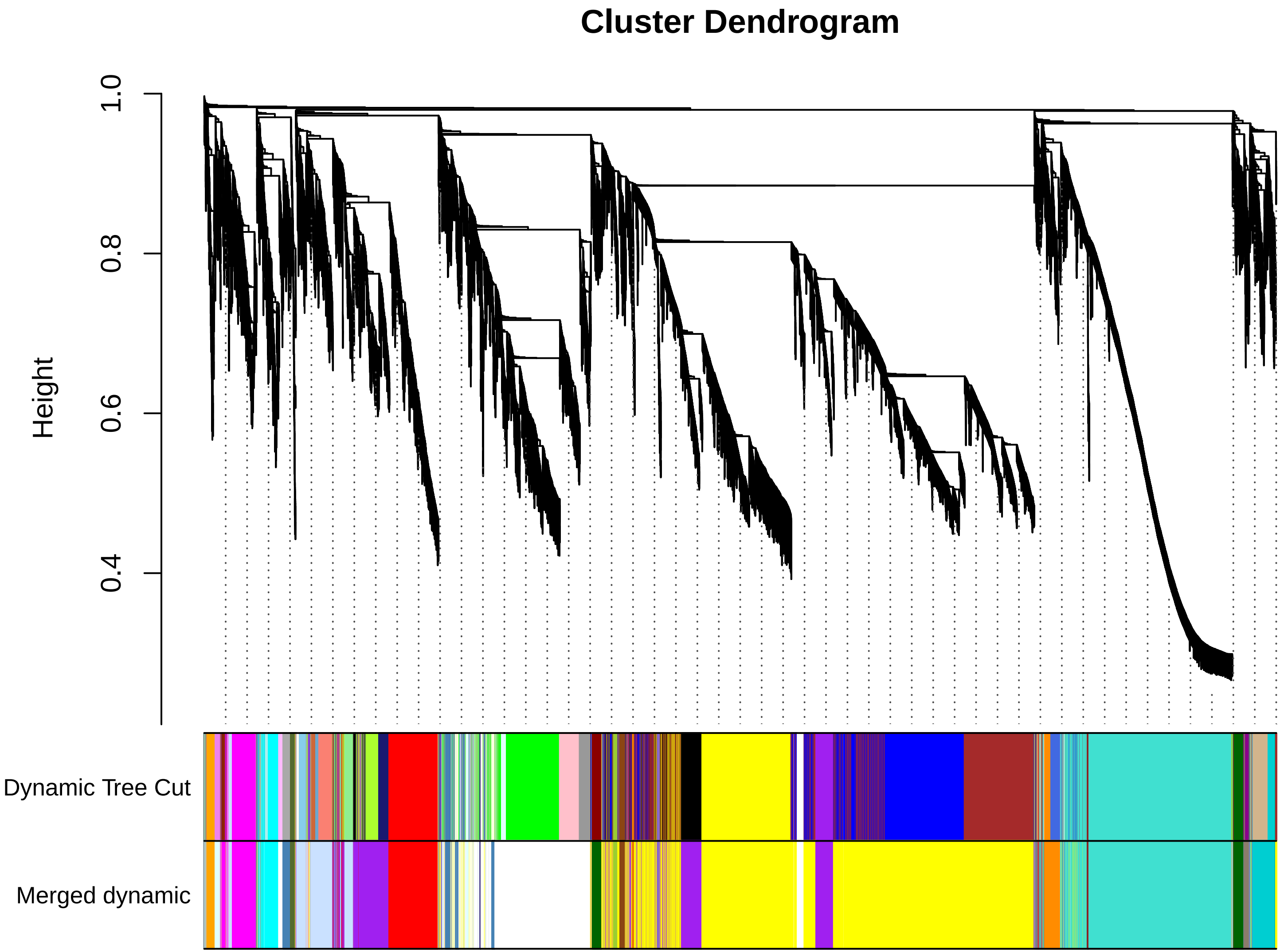

Cluster Dendrogram
Height
1.0
0.8
0.6
0.4
Dynamic Tree Cut
Merged dynamic
