# Supplementary material for: Integrated RNA-seq and small RNA-seq profiling reveals tissue-associated transcriptional programs and candidate miRNA–mRNA relationships across tea floral tissues
Source: Front Plant Sci. 2026 Sep 14;17:1941110. doi: 10.3389/fpls.2026.1941110 (PMC13617024; doi:10.3389/fpls.2026.1941110)
Supplement: Supplementary file 3 [file Image3.pdf]

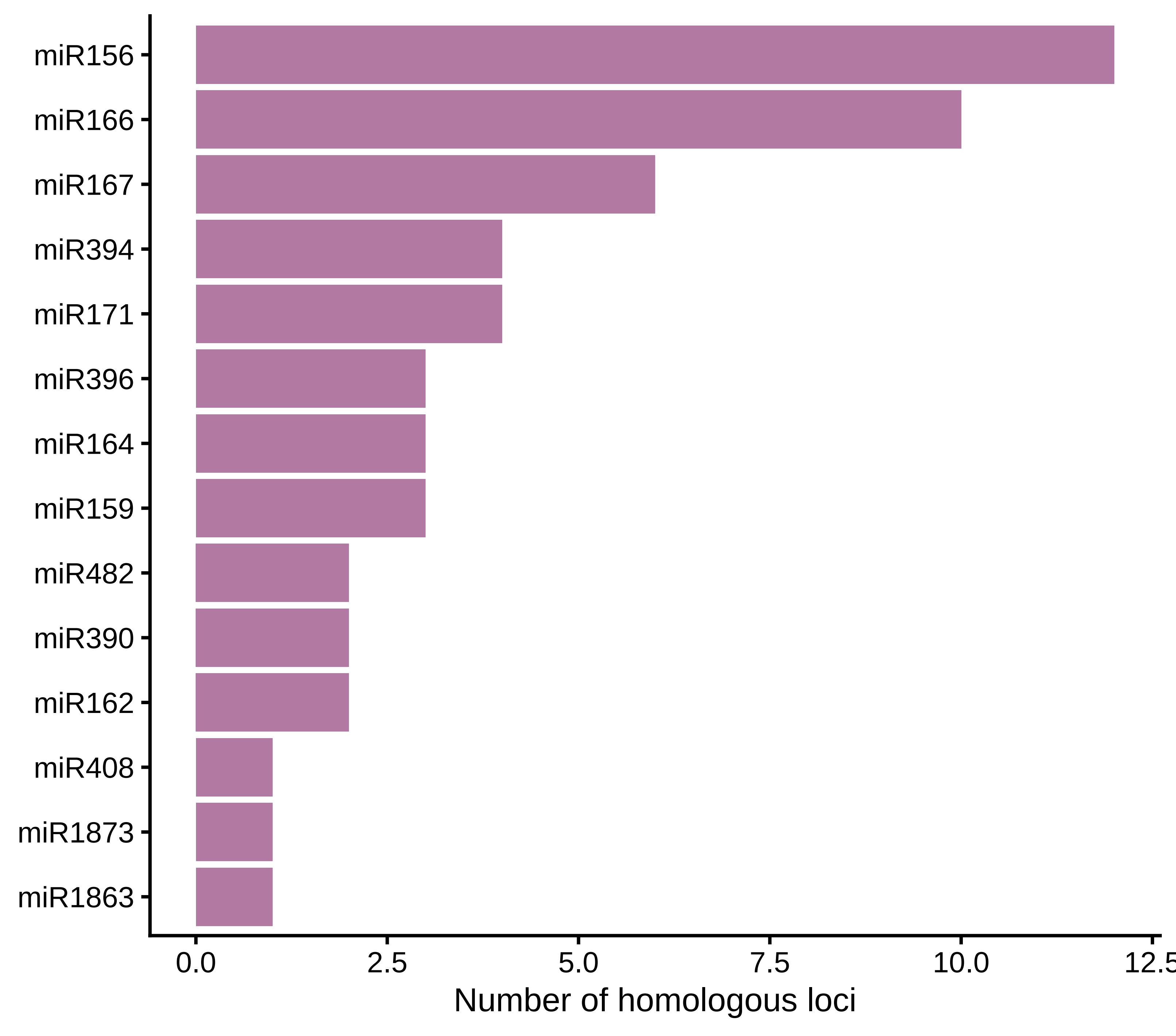
miR156
miR166
miR167
miR394
miR171
miR396
miR164
miR159
miR482
miR390
miR162
miR408
miR1873
miR1863
0.0
2.5
5.0
7.5
10.0
12.5
Number of homologous loci
