# Supplementary material for: Integrated RNA-seq and small RNA-seq profiling reveals tissue-associated transcriptional programs and candidate miRNA–mRNA relationships across tea floral tissues
Source: Front Plant Sci. 2026 Sep 14;17:1941110. doi: 10.3389/fpls.2026.1941110 (PMC13617024; doi:10.3389/fpls.2026.1941110)
Supplement: Supplementary file 4 [file Image4.pdf]

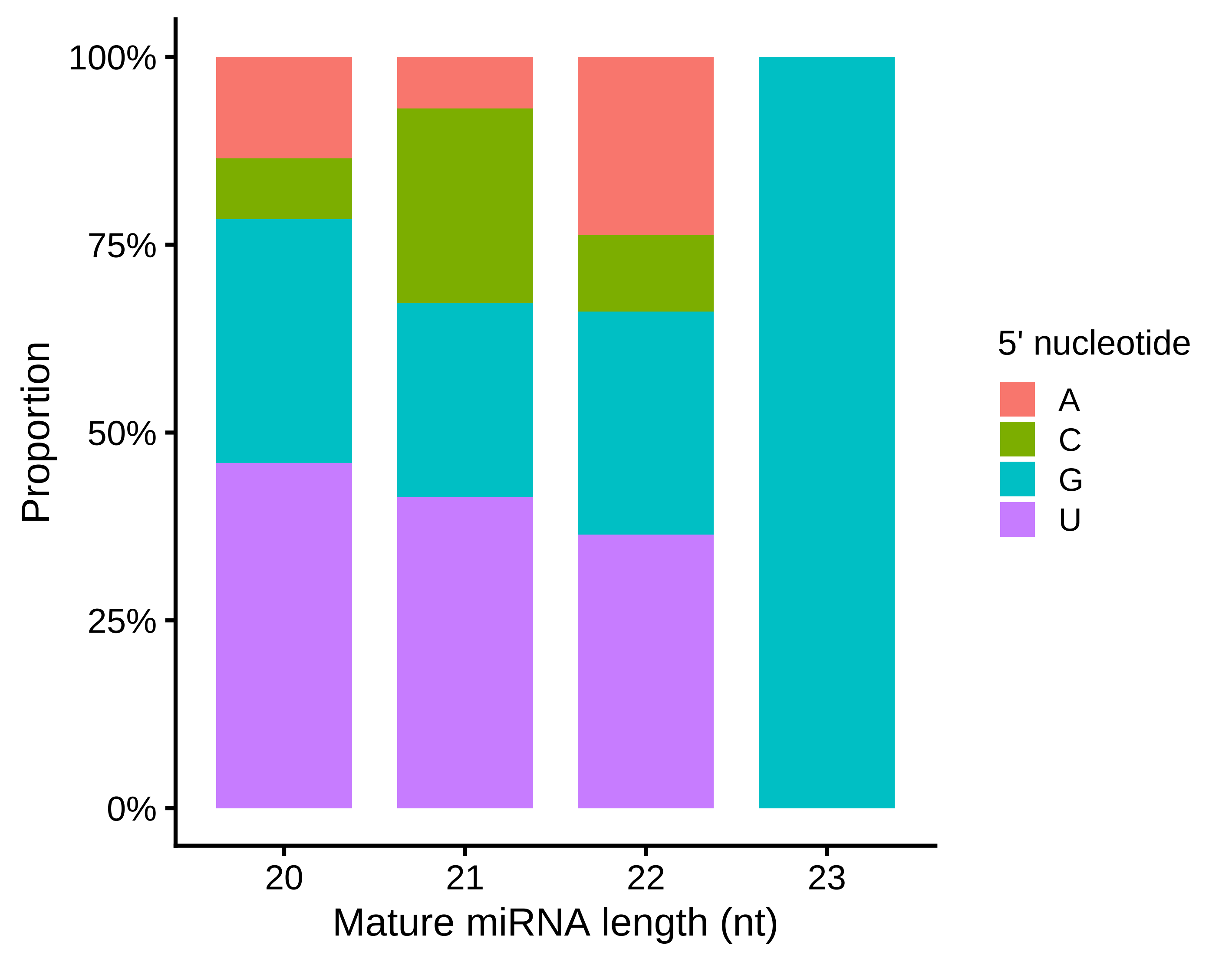
100%
75%
50%
25%
0%
Proportion
20
21
22
23
Mature miRNA length (nt)
5' nucleotide
A
C
G
U
